# An efficacy study of a combined parent and teacher management training programme for children with ADHD

**DOI:** 10.3109/08039488.2011.641587

**Published:** 2011-12-12

**Authors:** Monica Östberg, Ann-Margret Rydell

**Affiliations:** 1 Department of Women's and Children's Health, University Hospital of Uppsala, Uppsala, Sweden; 2 Department of Psychology, Uppsala University, Uppsala, Sweden

**Keywords:** ADHD, Intervention, ODD, Public health setting, Randomization

## Abstract

*Background:* Several parent training programmes and behavioural teacher training programmes built on learning theory have been developed for problem prevention and treatment of attention-deficit/hyperactivity disorder (ADHD) and/or oppositional defiant disorder (ODD) during the last few decades. Group format has often been used for parent training but single-subject designs are more common in teacher training. More studies have focussed on pre-school children than on older children, and a minority have been conducted in public mental health settings. *Aim:* This study aimed to evaluate a combined parent and teacher manual-based group training programme for children with ADHD conducted by the staff at a child and adolescent psychiatric clinic in Sweden. *Method:* The intervention was a modified version of Barkley's programme. Children were randomized to an Intervention or a Control group. Sixty-one parents and 68 teachers answered questions about ADHD and ODD symptoms, and about behavioural problems when the study started and at a 3-month follow-up. *Results:* Results showed that the intervention resulted in a reduction of the number of children who met DSM-IV criteria for ADHD and/or ODD. Effects were more pronounced in the home setting than in the school setting, and were further accentuated when both parents and teachers of the same child took part in the intervention. Teachers with more problematic classroom situations benefited most from the intervention. *Conclusion:* The programme, “Strategies in Everyday Life”, has, in a regular clinical setting, demonstrated promising effects on children's disruptive behaviour, and a clinical implication was to recommend involving both parents and teachers in the programme.

This paper reports on a randomized clinical study evaluating the effi cacy of a manual-based combined parent and teacher management training programme for children with attention-defi cit/hyperactivity disorder (ADHD) conducted in a public mental health setting.

ADHD is characterized by severe and persistent impulsivity, inattention and over-activity, affects 3–7% of school-aged children and is more common in boys than in girls with ratios of 1/6–10 in referred samples (1–3). A genetic predisposition as one factor behind ADHD is well documented (2–4).

ADHD is associated with high rates of comorbid disorders such as oppositional defi ant disorder (ODD) and conduct disorder (CD), as well as with internalizing problems, learning disorders and peer problems. Associated problems tend to be especially pronounced in children with comorbid disruptive disorders. Parents of afflicted children tend to regard themselves as less competent, to have more relationship problems and use more negative parenting strategies, compared with parents of non-afflicted children. ADHD problems are predictive of later poor adaptation and place costly demands on medical, psychological and societal resources (2–5).

Several parent training programmes (BPT) built on learning theory have been developed for problem prevention and treatment of ADHD and/or ODD during the last few decades (for reviews see references (6–8)). Some programmes have been in the format of individual contacts with families, but often a group format has been used. Programmes share features such as training in reinforcement and problem-solving strategies, promotion of positive parent – child interactions and of emotional communication (7). Behavioural teacher training (BTT) programmes have often been conducted with single-subject designs, and results from the few randomized controlled trial studies have been mixed (6, 8, 9). The positive effects of psycho-stimulant treatment for ADHD children is well documented, but a combination of pharmacological therapy and psychosocial or behavioural modifi cation treatment seems most effective (10). The behavioural treatment in the above study (10) was intense, and involved both the parents and the child's school into the programme.

More studies have reported on interventions for preschool children than for older children (11), and only a minority, about 4% (12), of intervention studies have been conducted in public mental health settings, which is a drawback, since results obtained under rigorous research conditions could be hard to generalize to everyday clinical practice (13).

## Aims

This study aimed to evaluate a combined parent and teacher training programme conducted by the staff at a child and adolescent psychiatric clinic in Sweden. As this programme has not been scientifically evaluated, we consider this an efficacy study. The children had verified attention and hyperactivity difficulties. Programme content was comparable with those of programmes evaluated in other countries, but had been adapted to suit Swedish parents. Programmes must be tried and evaluated in the cultural context where they are used. As there are mixed findings regarding the effect of problem severity on outcomes (6, 8, 13–16), we studied the severity of the child's problem as a possible moderator. Another severity aspect, that of parents' and teachers' perceptions of the burden of the child's problems in terms of conflicts with the child and control over the child's behaviour, was also examined as a possible moderator.

We investigated whether BPT and BTT were effective treatments for referred children with ADHD. This was tested separately for BPT and BTT, as well as in combination. We hypothesized that effects on outcomes would be more pronounced when both parents and teachers of the same child had participated in the intervention.

## Methods

The study was approved by the local ethics committee (Dnr 2005:359), and has been performed in accordance with the ethical standards laid down in the 1964 Declaration of Helsinki. All persons in the study have given their informed consent to inclusion in the study, and full anonymity was granted.

### Setting, intervention and procedure

The setting was the four units of a mid-Sweden County Child and Adolescent Psychiatric Clinic. The intervention is routinely offered to parents and teachers to children with ADHD problems, and is a slightly modified version of Barkley's parent training programme (17) adapted to Swedish circumstances and conditions. A parallel and similar programme for teachers was constructed with the goal to address the child's two major contexts, home and school at the same time. The modifications were: 1) “time-out” for unwanted behaviour was excluded, as earlier evaluations had shown that parents were not capable of carrying through the time-out, resulting in more frequent everyday conflicts; 2) home assignments were based on the problems that parents and teachers had experienced and reported on. This resulted in a stronger motivation to do the assignments, but also that the problem-solving training in the programme was extended. Other main ideas from Barkley's programme were kept and the aim was to give “tools” to parents and teachers and to form “Strategies in Everyday Life” (which is also the name of the programme) in order to help the child. The intervention is manual based, as is training for group leaders (18, 19). Parents meet for 10 weekly 2-h sessions, and teachers meet for eight sessions, with parents/teachers of about eight children per group. The sessions focus on information about neuropsychiatric problems and on participants learning to use reinforcements, to solve problems and to communicate with the child. Home assignments and discussions of these are part of the programme, and a structure for the co-operation between home and school is formed.

Parents of 7–10-year-old patients with neuropsychiatric problems without mental retardation were consecutively invited to take part in the intervention. Across 1½ years, six groups were recruitedx. Parents who did not wish to participate were invited to join a group outside the study. Participating parents agreed to be randomized to intervention directly (Intervention group), or to intervention after completion of the study (Control group). Parental consent to collect information from the child's medical record was obtained from all except one family, and following consent from parents (consent was obtained from all families), the child's teachers were invited to parallel groups. Six well-trained group-leaders, two per group in varying constellations, performed the study intervention. Both parents and two of the child's teachers were invited.

Parents completed questionnaires about their child's ADHD and ODD behaviours and matched pairs, based on age, gender and the level of ADHD and ODD symptoms, were formed. One child/pair was randomized to each group. Parents and teachers completed questionnaires pre-(Tl) and post-study (T2) and at a 3-month follow-up (T3). For children with two informants from home or school, data from one parent and one teacher was selected for the analyses. Parents should be a biological parent or a permanent foster parent, have participated in five or more group sessions (Intervention group), have data from T1 and from T2 and/or T3 and have the higher ADHD and ODD ratings at T1 of the parents. Teachers should be the child's main teacher, have the higher participation rate of the two teachers and having participated in four or more of the group sessions (Intervention group) and have data from T1 and from T2 and/or T3.

### Participants

**Fig. 1:**
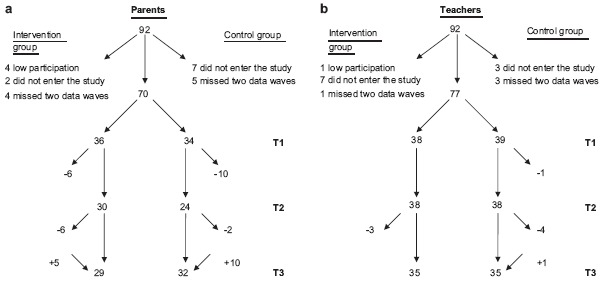
Flowchart for the three data waves (T1, T2, T3) for (a) parents and (b) teachers.

Families of 92 children agreed to enter the study. However, as seen by the flowcharts in Fig. 1, there was attrition in both groups and in parent and teacher participation. Twenty-two parents were excluded from the study (Fig. 1a). Attrition was related to family stressors. Teachers of 15 children were excluded (Fig. 1b). Attrition was related to time shortage or the child's change of school during the study. Please note that parents/teachers in some cases did not participate at T2 but returned to the study at T3. Most analyses are based on data from T1 and T3: 61 parents and 68 teachers. Parents in the Intervention group participated in mean = 8.4 group sessions standard deviation ± 1.3 group sessions, and for teachers' participation was mean = 6.7 ±1.1 sessions.

Ninety-three per cent of the children were diagnosed with ADHD, and the rest had similar problems but were not yet diagnosed. These children had recently been referred to the Child and Adolescent Psychiatric Clinic and were still in the process of being evaluated for a diagnosis. ADHD diagnoses were made by the child psychiatrist. The psychiatric evaluation encompassed information from a medical examination made at the visit to the clinic and information from parents and school including IQ testing, questionnaires screening for neuropsychiatric problems (the Five to Fifteen, FTF; 20), the Autism Spectrum Screening Questionnaire (ASSQ; 21) and the ADHD symptom questionnaire (SNAP-IV; 1).

Comorbid diagnoses to ADHD were Asperger's syndrome (four children) and tics (three children); 25 children (86%) in the Intervention group and 24 children (77%) in the Control group were on medication with stimulants during the intervention time. The groups did not differ regarding diagnoses or medication (*P* > 0.05). Information from the medical records revealed that beside the intervention given to the Intervention group, very few children/families in any of the two groups received other interventions than monitoring medication. Notes in the medical records revealed general counselling to four families, one in the Intervention group and three in the Control group. Three children in each group had been placed in special education groups or received other form of extra help at school.

The groups did not differ in socio-demographic characteristics (Table 1), nor in ethnicity or family composition, *P* > 0.05. Ninety-two per cent of the mothers and 87% of the fathers were born in Sweden. Sixty-two per cent of the children lived with both parents; the rest alternated between parents or lived with one parent, and one child in each group lived with foster parents. Forty-nine per cent of the parents (no group difference, *P* > 0.05) had participated in parent groups geared towards child behaviour problems, but only for two families were pervious interventions comparable with the present one.

**Table 1:** Demographic characteristics of parents and children.

	Group 1 (*n* = 29)			Group 2 (*n* = 32)			Difference between groups	
	Mean	*s*	*n* (%)	Mean	*s*	*n* (%)	*t* χ^2^	*P*
Child								
Age (years)	11.1	2.1		10.8	1.8		0.66	.51
Boys			25 (86)			26 (81)	0.27	.60
Parents							0.04	.98
Mother			22 (76)			25 (78)		
Father			6 (21)			6 (19)		
Foster parent			1 (3)			1 (3)		
Mother's age (years)	39.6	5.3		38.2	5.9		0.99	.33
Father's age (years)	41.7	5.9		41.1	7.3		0.35	.73
Mother's education							0.14	.93
Compulsory 9 years			3 (10)			3 (9)		
2–3 years high school			12 (42)			12 (38)		
College			14 (48)			17 (53)		
Father's education							1.62	0.44
Compulsory 9 years			5 (18)			10 (31)		
2–3 years high school			10 (36)			11 (34)		
College			13 (46)			11 (34)		

*s*, standard deviation.

Group 1 = Intervention group; Group 2 = Control group.

School staff was class teachers, 85% and 74% in the two groups, and teachers' aid, *P* < 0.05. Age (mean = 44 years) and professional experience (mean = 15 years) did not differ between groups, *P* > 0.05. Participants had taught the child for at least 1 year before the study. Three teachers in each group were replaced at T3 because of the child's change of class.

### Instruments

*ADHD symptoms* were assessed by the ADHD Rating Scale, which reflects the 18 DSM-IV criteria and is extensively used in research (1, 22). Responses are given on a four-step scale from 0 = never/rarely to 3 = very often and scores > 2 on individual items considered the symptom being present. Criteria for the DSM-IV ADHD subtypes in home and school, respectively, followed the APA manual (1).

*ODD symptoms* were measured by the eight DSM-IV criteria (1). Response format and scoring was as above. Criteria for an ODD diagnosis followed the APA (1).

Parent and teacher ratings of ADHD and ODD symptoms at T1 were added and two groups in home and school, respectively, were formed based on median split, representing one group with high and one group with low symptom levels.

The *Strengths and Difficulties Questionnaire (SDQ-SWE)* was used as an additional assessment of problem behaviours. The SDQ has been validated in Sweden and has adequate psychometric properties (23, 24). Ratings were made on five-step response scales, from 1 = “does not apply at all” to 5 = “applies very well”. The five-item scales for emotional symptoms and the total 20-item scale score were used.

*Perceived burden* was measured with the 10-item Locus of Control Scale (25), which taps parents' and teachers' experience of control of the child's behaviour. Conflicts at school were measured by the 13-item Pianta Conflict Scale (26) and conflicts in the home were measured by 12 newly constructed items paralleling the Pianta items. Ratings were made on five-step scales, 1 = “does not apply at all” and 5 = “applies very well”. The aggregated parent and teacher measures of perceived control and conflicts at T1 were used to form two groups based on median split in home and school, respectively, representing high and low perceived burden.

### Statistical analyses

Analyses were performed using the Statistical Analyses System (SAS®). Differences between groups were analysed by means of chi-square tests, *t*-tests and with two-way (group × time) repeated-measures analysis of variance (ANOVA). To explore significant interaction effects, Cohen's *d* (27) was calculated. In accordance with convention, *d* ≥ 0.80 was regarded as a large effect, *d* ≥ 0.50 as a medium effect and *d* ≥ 0.20 as a small effect. Moderator effects were studied by 2 (group) × 2 (time) × 2 (moderator variables with two levels) repeated-measures ANOVAs.

## Results

Means and standard deviations for parents' and teachers' ratings on number of ADHD and ODD symptoms, and mean scores and standard deviations for the SDQ scales, the Locus of Control scales, the Conflicts at home and at school scales, and for the aggregated measure of perceived control at T1, T2 and T3 are presented in Table 2. The groups did not differ on any parent or teacher rated outcome variable at T1, *P* > 0.05. All results below have been controlled for outliers.

**Table 2:** Means and standard deviations at T1, T2 and T3 in both groups for parents' and teachers' ratings on number of attention-deficit/hyperactivity disorder (ADHD) and oppositional defiant disorder (ODD) symptoms, scales for the Strengths and Difficulties Questionnaire (SDQ), Locus of Control, Conflicts at home and at school, and for the aggregated measure of Perceived burden.

	Parents			Teachers		
	T1 *n* = 36/34 Mean (*s*)	T2 *n* = 30/24 Mean (*s*)	T3 *n* = 29/32 Mean (*s*)	T1 *n* = 38/39 Mean (*s*)	T2 *n* = 37/38 Mean (*s*)	T3 *n* = 34/34 Mean (*s*)
Symptom ADHD-C						
Group 1	10.8 (3.8)	9.1 (4.5)	7.7 (4.7)	8.1 (5.2)	7.7 (6.3)	7.7 (5.7)
Group 2	10.7 (4.7)	9.8 (6.0)	10.1 (5.3)	10.2 (5.4)	9.4 (6.3)	9.4 (5.4)
Symptom ADHD-HI						
Group 1	4.5 (2.8)	3.9 (2.8)	3.2 (2.7)	3.5 (2.9)	3.3 (3.2)	3.6 (2.9)
Group 2	4.9 (2.8)	4.7 (3.2)	4.8 (3.0)	4.4 (3.2)	4.0 (3.6)	3.9 (3.3)
Symptom ADHD-IA						
Group 1	6.3 (2.1)	5.2 (2.3)	4.5 (2.5)	4.6 (3.1)	4.4 (3.5)	4.1 (3.4)
Group 2	5.8 (2.6)	5.1 (3.1)	5.3 (2.9)	5.8 (2.7)	5.5 (3.3)	5.5 (2.6)
Symptom ODD						
Group 1	2.8 (2.3)	2.4 (2.2)	1.7 (1.9)	2.2 (2.6)	1.5 (2.4)	1.1 (1.9)
Group 2	3.6 (2.5)	3.2 (2.5)	3.3 (2.6)	3.0 (3.1)	2.7 (3.1)	2.6 (3.2)
SDQ-total						
Group 1	2.8 (0.5)	2.7 (0.5)	2.5 (0.6)	2.7 (0.5)	2.5 (0.6)	2.5 (0.5)
Group 2	2.7 (0.5)	2.7 (0.6)	2.7 (0.6)	2.8 (0.6)	2.8 (0.6)	2.9 (0.6)
SDQ-emotional symptoms						
Group 1	2.6 (0.9)	2.5 (0.9)	2.1 (0.8)	2.4 (0.9)	2.1 (0.9)	2.1 (0.9)
Group 2	2.4 (1.0)	2.3 (0.9)	2.5 (0.9)	2.4 (0.9)	2.4 (0.9)	2.5 (1.1)
Locus of control						
Group 1	3.4 (0.8)	3.4 (0.6)	3.4 (0.9)	3.7 (0.8)	3.7 (0.8)	4.0 (0.7)
Group 2	3.3 (0.8)	3.2 (0.8)	3.3 (0.8)	3.6 (0.9)	3.7 (0.9)	3.6 (0.9)
Conflicts home/school						
Group 1	2.8 (0.8)	2.8 (1.0)	2.5 (0.8)	2.3 (0.8)	2.2 (0.8)	2.0 (0.7)
Group 2	2.9 (0.8)	2.9 (0.9)	2.9 (0.9)	2.4 (0.9)	2.3 (0.9)	2.3 (0.9)
Perceived burden						
Group 1	2.74 (0.72)	2.77 (63)	2.60 (0.78	2.24 (0.78)	2.23 (0.77)	2.14 (0.70)
Group 2	2.79 (0.73)	2.84 (0.76)	2.76 (0.76)	2.41 (0.87)	2.32 (0.84)	2.37 (0.83)

*s*, standard deviation.

Group 1 = Intervention group; Group 2 = Control group.

### Effects of the intervention

As regards assessments at three time points, there was one significant interaction effect on parent ratings of the SDQ-total, *F* = 4.74, *P* < 0.05. The problematic behaviours were reduced only in the Intervention group, showing small effect sizes. As attrition was fairly large among parents at T2, we analysed T1 to T3 results to obtain more power. A reduction of parent-rated ADHD symptoms, i.e. ADHD-C, ADHD-HI and ADHD-IA, and in problem levels of the two SDQ scales was evident in the Intervention group only, *F* = 4.05–8.95, *P* < 0.05 to *P* < 0.01, with medium to large effect sizes. In the teacher ratings, the Intervention group reduced the emotional problems more than the Control group, *F* = 4.29, *P* < 0.05.

### Clinical significance

A clinically significant effect of the intervention would be reduced numbers of children fulfilling the DSM-IV criteria for ADHD and/or ODD in the Intervention group. According to parents' assessments, significantly fewer children in the Intervention group than in the Control group reached the criteria for ADHD-C, ADHD-HI and ODD at T3, while there were no differences at T1 (Table 3). More children in the Intervention group than in the Control group reached criteria for ADHD-IA at T1, but at T3 this difference had vanished. Corresponding analyses for teachers showed a significantly reduced number of children fulfilling criteria for ODD in the Intervention group at T3 compared with the Control group (χ^2^ = 4.66, *P* < 0.05), but no differences at T1.

**Table 3:** Number of children reaching the criteria for diagnosis of attention-deficit/hyperactivity disorder (ADHD) and oppositional defiant disorder (ODD), according to parents' assessments at T1 and T3.

	Parents, reaching criteria at T1, *n* = 61 (29/32)			Parents, reaching criteria at T3, *n* = 61 (29/32)		
	Yes	No	χ^2^	Yes	No	χ^2^
ADHD-C			1.19			4.00*
Group 1	11	18		5	24	
Group 2	8	24		13	19	
ADHD-HI			0.11			5.36*
Group 1	13	16		7	22	
Group 2	13	19		17	15	
ADHD-IA			4.10*			0.46
Group 1	21	8		12	17	
Group 2	15	17		16	16	
ODD			0.21			6.81**
Group 1	11	18		6	23	
Group 2	14	18		17	15	

* *P* < 0.05

** *P* < ; Group 1 = Intervention group; Group 2 = Control group.

### Effects when parent and teacher of the same child took part in the intervention

The hypothesis that effects should be stronger when both parent and teacher of the same child took part in the intervention, was tested on the 45 (23/22) children whose parents and teachers participated and who had data from T1 and T3. Aggregated parent and teacher measures were used in these analyses. Interaction effects were found for all variables except for ADHD-HI (Table 4). Problem levels were reduced in the Intervention group, but not in the Control group.

**Table 4:** Analyses of variance (ANOVAs) with repeated measure on the mean of parents' and teachers' assessments of the same child in the two groups at T1 and T3 (*n* = 45; 23/22).

	T1, mean (*s*)	T3 mean (*s*)	Interaction, time × group, *F*	Effect size, T1–T3, *d*
Symptom ADHD-C			4.23*	
Group 1	9.1 (3.3)	7.3 (4.0)		0.49
Group 2	10.4 (4.1)	10.4 (4.0)		0.01
Symptom ADHD-HI			2.09	
Group 1	3.8 (2.2)	3.3 (2.3)		0.24
Group 2	4.6 (2.4)	4.8 (2.3)		0.08
Symptom ADHD-IA			4.07*	
Group 1	5.3 (2.0)	4.0 (2.2)		0.59
Group 2	5.7 (2.2)	5.6 (2.0)		0.07
Symptom ODD			12.56***	
Group 1	2.4 (1.9)	1.1 (1.4)		0.75
Group 2	3.5 (2.4)	3.5 (2.4)		0.02
SDQ-total			11.70**	
Group 1	2.6 (0.4)	2.4 (0.4)		0.53
Group 2	2.8 (0.4)	2.9 (0.4)		0.25
SDQ-emotional symptoms					6.53*	
Group 1	2.3 (0.8)	2.0 (0.7)		0.48
Group 2	2.5 (1.0)	2.5 (0.8)		0.09

* *P* < 0.05

** *P* < 0.01

*** *P* < 0.001; Group 1 = Intervention group; Group 2 = Control group.

### Moderator analyses

*Moderator analyses* were performed on T1 to T3 ratings. Symptom levels at T1 had effects in both home and school. For SDQ-total parent ratings, at high symptom levels, parents in the intervention group reported lower ratings at T3 than at T1, *d* = 0.89, whereas parents in the Control group reported somewhat higher ratings, *d* = 0.20. These effects were not evident if the symptom load was low, *F* = 3.38, *P* < 0.10. Similarly, the interaction on teacher ratings was significant regarding the SDQ-emo-tional scale, *F* = 4.44, *P* < 0.05. At high symptom load, the problem level was reduced in the Intervention group, *d* = 0.58, but had increased in the Control group, *d* = 0.42, which was not the case at low symptom load. There was one effect of perceived burden, in teacher ratings regarding group effects on the reduction of ODD symptoms, *F* = 5.81, *P* < 0.05. If burden was high, the effect size was large in the Intervention group, *d* = 1.15 and small in the Control group, *d = 0A9,* whereas no such effect was found in the low burden group.

## Discussion

In this randomized efficacy study of a parallel parent and teacher intervention, parents in the Intervention group reported a reduction of their child's ADHD symptoms and behavioural problems at follow-up. Furthermore, according to parents, and regarding ODD to teachers, the intervention resulted in a reduction of the number of children who met DSM-IV criteria for ADHD and/or ODD. When parents and teachers to the same child took part in the intervention, significant effects were registered for most outcomes. Generally, intervention effects seemed stronger in the home setting than at school. However, teachers describing the children as highly symptomatic at start, and teachers experiencing a high burden in dealing with the child, reported the intervention to be more effective than teachers describing lower problem loads.

Our results rely on T1 to T3 comparisons, as there was a large attrition among parents at T2. However, the pronounced effects compared with those including post-assessments may also indicate that it takes time to implement new strategies. Effect sizes of interventions in parental assessments of child problems were comparable with those of other studies of school-aged children with ADHD problems (7, 8, 13), and regarding teachers somewhat lower, or in the same range (8, 9). As regards clinical significance, parents reported more extended intervention effects than teachers.

The children in this study had significant disruptive problems, and a majority was on stimulant medication. This might partly explain the smaller effects reported by teachers than by parents. Effects of stimulant medication, being mostly administered in the mornings, are probably more prominent during the school day than in the evening when the effects are fading off. Furthermore, parents may be more able to try new strategies with the child at home than teachers who are responsible for a classroom. Smaller intervention effects for BTT compared with BPT have been reported also in a recent review of evidence-based treatments for ADHD (8). In this light, one should reflect upon the positive effect for teachers of high perceived burden in handling the child. Teachers who reported a low level of control and many conflicts in the classroom situation seemed to have benefited the most from the intervention, expressed in reduced levels of ODD symptoms at follow-up. Maybe a severely problematic classroom situation motivated them to adopt the new strategies. Thus, in performing interventions geared towards teachers, investigators would be well advised to assess how teachers perceive their classroom situation. However, a higher symptom level boosted intervention response for both parents and teachers. There is mixed support (6, 8) for moderating the effects of problem severity in the literature, but our results add to those identifying such effects (15, 16). The somewhat fewer moderation effects in parent than in teacher ratings may indicate that parents of children with ADHD are always motivated to try to change things for the better, and are therefore less affected by the day-to-day difficulties with the child than are teachers.

A hallmark of the intervention is its ambition to address simultaneously the child's two major contexts. As expected, effects were more pronounced when both parents and teachers had taken part in the intervention. The effects of the intervention were no larger, but as we found significant intervention effects for most symptom variables, they were more comprehensive than in the separate analyses on parents and teachers. Obviously, it can be worth the effort to direct interventions to a broad spectrum of the child's everyday life.

Concerns about identification of effective components of behavioural interventions have been raised (6, 8). This study was not designed to shed light on these questions. However, in addition to the results previously reported, user satisfaction was assessed. The majority of parents and teachers were highly satisfied with the intervention, but several respondents indicated that training in solving problematic situations and in co-operation between home and school should be given more attention in the programme.

A strength of the study is that the intervention was delivered in routine care. Controlled studies with a very strict methodology generally report higher effect sizes, but the results can be difficult to generalize to standard settings (13). We showed that clinically significant changes could be obtained within routine clinical practice. A drawback of the study is the relatively high attrition. Although parents were motivated to participate, several missed one or two data waves. Bearing in mind that the sample was recruited while in child psychiatric care, this was not altogether unexpected. For parents, the main motivation to be in the study was probably to get help, and not to fill out questionnaires. It should also be noted that schools were sometimes reluctant to allocate teacher time to the interventions. However, the Intervention and the Control groups were equally afflicted, thus the results are not affected by attrition. Also, we had no information about the proportion of children with medication at follow-up (T3). However, it is highly unlikely that our results could be ascribed to any changes in medication. The majority of children was on medication and monitored. Finally, we relied only on parents' and teachers' perceptions of the children. Independent measures, such as observations of child behaviour, would have strengthened the study.

## Conclusions

The combined parent and teacher training programme “Strategies in Everyday Life” demonstrated clinically significant effects on several aspects of child behaviour especially in the home context. The children in this study had clinically identified problems with ADHD, most of them were on stimulant medication and half of their parents had earlier taken part in other group training programmes. They had manifested problems and were in middle childhood. In order to influence the conditions for the children in several respects, both parents and teachers took part in the intervention, and effects were found to be more prominent when both parties underwent training. This is a hallmark of the programme, and we recommend that caregivers to put in efforts to involve both contexts. Furthermore, our results demonstrated that teachers who reported a low level of control and more classroom conflicts benefited most from the programme. To summarize, for parents and teachers who followed through with the intervention, the “Strategies in Everyday Life” programme has, in a routine care setting, demonstrated several effects on children's disruptive behaviour, especially when parallel sessions for parents and teachers took place. In light of the serious and persistent problems of the children in this study, these results are promising.
